# Preoperative pelvic floor muscle diameter as a predictor of postoperative urinary incontinence in robotic‐assisted laparoscopic total prostatectomy

**DOI:** 10.1002/bco2.70001

**Published:** 2025-03-03

**Authors:** Sho Hashimoto, Daisuke Obinata, Hideaki Uchida, Shigeyuki Arakawa, Yuki Inagaki, Ken Nakahara, Tsuyoshi Yoshizawa, Junichi Mochida, Kenya Yamaguchi, Satoru Takahashi

**Affiliations:** ^1^ Department of Urology Nihon University School of Medicine Tokyo Japan

**Keywords:** obturator internus muscle, pelvic floor muscle, prostate cancer, pubococcygeal muscle, robotic‐assisted laparoscopic total prostatectomy, urinary incontinence

## Abstract

**Objective:**

To assess the impact of preoperative pelvic floor muscle thickness on the early recovery of urinary continence following robot‐assisted radical prostatectomy (RARP).

**Patients and Methods:**

A retrospective study was conducted on 114 patients who underwent RARP at our institution between January 2019 and March 2021. Patients included were either confirmed to be pad‐free or using only safety pads postoperatively or those with persistent incontinence, with a follow‐up period of at least 6 months. Patient characteristics, perioperative outcomes, and pelvic floor muscle diameters were analysed. Preoperative magnetic resonance imaging or computed tomography was used to measure the diameters of the pelvic floor muscles, including the obturator internus and pubococcygeus muscles.

**Results:**

The median patient age was 68 years (interquartile ranges [IQR]: 64–72 years), with a median Prostate‐Specific Antigen (PSA) of 7.8 ng/ml (IQR: 5.4–10.6 ng/ml). The median prostate volume was 26.6 ml (IQR: 23–35 ml) in the early recovery group and 29 ml (IQR: 19.5–40 ml) in the delayed recovery group. The median time to continence recovery was 5.0 months (95% confidence interval: 4.2–5.7 months), with an incontinence resolution rate of 85.1%. Significant associations were found between the thicknesses of the obturator internus (p = 0.025) and pubococcygeal muscles (p = 0.004) and early continence recovery. Nerve‐sparing procedures were also associated with faster recovery (p = 0.016). Multivariate analysis identified the thickness of both muscles as independent predictors of early continence recovery.

**Conclusion:**

Preoperative evaluation of pelvic floor muscle thickness, particularly the obturator internus and pubococcygeal muscles, may help predict early postoperative urinary continence recovery in patients undergoing RARP. Preoperative pelvic floor muscle exercises to strengthen these muscles could improve the postoperative outcomes.

## 1 INTRODUCTION

Prostate cancer is a common type of cancer among men, with a steadily increasing incidence.^1^ In recent years, advances in prostate‐specific antigen (PSA) screening have resulted in an increase in early diagnosis.^2^ However, the diagnosis of prostate cancer has advanced beyond confirmation of the disease and has become a pivotal factor that influences treatment decisions and prognosis.^2^

Radical prostatectomy (RP) is a widely accepted treatment for prostate cancer, and robot‐assisted RP (RARP) is widely used in clinical practice due to its exceptional surgical approach.^3^ Despite this, post‐RARP urinary incontinence (UI) remains a challenge. The negative impact of UI on the patients' quality of life is significant and should not be overlooked.^4^ In particular, postoperative UI can severely restrict a patient's daily activities and potentially cause social and psychological discomfort.^5^ Recent research revealed no substantial difference in postoperative urinary incontinence at 12 months between robotic‐assisted laparoscopic prostatectomy (RARP) and open retropubic RP, indicating that there is no functional advantage associated with the surgical techniques employed.^6^ Consequently, the prevention and improvement of UI have become crucial aspects of prostate cancer treatment.

Examining the predictive factors of post‐RARP UI is important. Factors influencing UI include age, obesity, overactive bladder, muscle underactivity, surgical proficiency and positive margins on pathological findings.^7^, ^8^

These factors and preoperative anatomical features are also implicated in the occurrence and recovery of UI.^7^ The preservation of pelvic floor muscle structure during RARP facilitates the early recovery of bladder control.^9^ Hence, the size of the pelvic floor muscles has a high potential as a predictive factor for UI.

Focusing on these anatomical features also strengthens the evidence for the benefits of preoperative pelvic floor muscle training interventions. By targeting these muscles preoperatively, patients may experience faster and more complete postoperative recovery of urinary continence.

This study aimed to investigate the relationship between pelvic floor muscle size and post‐RARP urinary incontinence levels in patients with prostate cancer.

## 2 PATIENTS AND METHODS

The study adhered to the Strengthening the Reporting of Observational Studies in Epidemiology guidelines.^10^ Ethical approval was obtained from the Institutional Review Board and the Research Ethics Committee of the Nihon University School of Medicine (Reference Code −190 611‐3). Informed consent was obtained through an opt‐out method, and patient details were anonymized.

**FIGURE 1:**
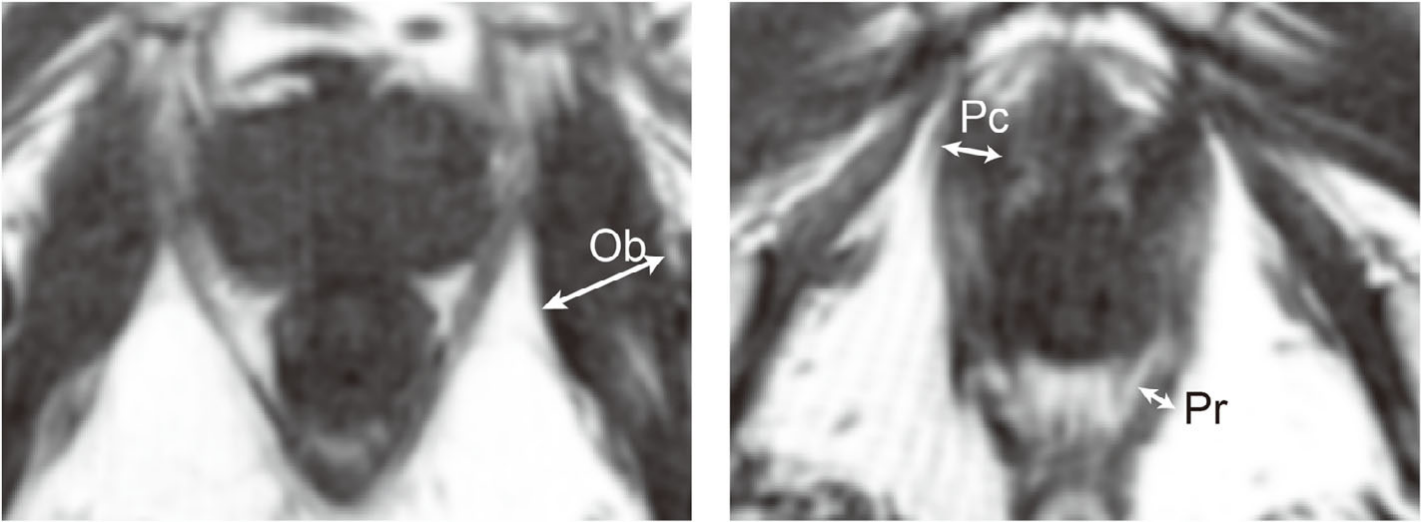
Measurement of the maximum diameter (indicated by arrows) of pelvic floor muscles on magnetic resonance imaging (MRI). Measurements were conducted in triplicates for each muscle to ensure precision. Ob, obturator internus muscle; Pc, pubococcygeus muscle; Pr, puborectalis muscle.

This study included 150 consecutive cases of RARP performed between January 2019 and March 2021. The inclusion criteria were as follows: patients without preoperative incontinence, those who underwent magnetic resonance imaging (MRI), and those confirmed to be pad‐free postoperatively via a questionnaire in our department. For patients who achieved continence recovery, the median follow‐up time at the point of recovery was 5 months. The exclusion criteria for this study encompassed patients with preoperative incontinence and those lacking preoperative continence evaluation. Furthermore, patients who did not undergo preoperative imaging studies were excluded. In cases where incontinence persisted, follow‐up was extended up to 24 months. Patients with persistent incontinence who were lost to follow‐up within 6 months were excluded. All patients were instructed in pelvic floor muscle exercises during the perioperative period, and all RARP procedures were performed using the da Vinci Surgical System. The standard procedure included nerve‐sparing and lymph node dissection when appropriate. Patient data were retrospectively reviewed from the medical records. The baseline characteristics, perioperative variables and postoperative outcomes (including incontinence status) were collected and analysed. Incontinence was defined as either pad‐free or using only a safety pad. Additionally, the maximal diameters of the obturator internus, pubococcygeus and puborectalis muscles were measured three times via MRI or computed tomography, and the average values were calculated (Figure 1). The measurements were conducted retrospectively by an experienced surgeon (H.U.) who was not involved in the surgical procedures during the study period and performed the measurements without prior knowledge of the surgical outcomes. Postoperative incontinence status was assessed during outpatient visits. Prior to the consultation, patients were interviewed by a nurse using a structured questionnaire, and additional confirmation was obtained during the physician's examination.

Statistical analyses were performed using Statistic Package for Social Sciences Software (IBM Japan, Tokyo, Japan) and GraphPad Prism for Mac 10 (GraphPad Software, Inc., La Jolla, CA, USA). Continuous variables are expressed as medians with interquartile ranges (IQR). The Mann–Whitney U test was used to compare continuous variables between the two groups. Categorical variables were analysed using the chi‐squared test. Receiver operating characteristic curve analysis was performed to assess the influence of pelvic floor muscle diameter on incontinence. The cutoff values for each muscle diameter were determined based on the data points that maximized the Kolmogorov–Smirnov statistic, identifying the optimal cutoff values for predicting incontinence. Significant predictors of incontinence were analysed using multivariate Cox proportional hazard analysis. A cumulative improvement in incontinence was constructed. Statistical significance was set at p < 0.05. significant.

## 3 RESULTS

**FIGURE 2:**
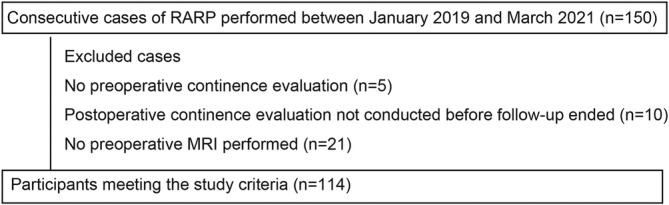
The study initially included 150 consecutive cases of robot‐assisted radical prostatectomy (RARP) performed between January 2019 and March 2021. Cases were excluded based on the following criteria: absence of preoperative continence evaluation (n = 5), postoperative continence evaluation not conducted prior to the conclusion of follow‐up (n = 10) and lack of MRI examination (n = 21). The final analysis comprised 114 patients.

In total, 114 patients met the eligibility criteria for this study (Figure 2). The patient characteristics are shown in Table 1. The median age is 68 years (IQR: 64–72 years), and the median PSA level at diagnosis is 7.8 ng/ml (IQR: 5.4–10.6 ng/ml) (Table 1). Twenty patients have diabetes, 41 have hypertension, 3 have a history of myocardial infarction and 4 have a history of neurological disorders. The Gleason score at diagnosis is six in 17 cases, seven in 58 cases and eight or higher in 42 cases (Table 1). The median time from MRI to surgery was 115 days (IQR: 83–169 days) (Table 1). Lymph node dissection is performed in 58 cases, and tumour involvement is found in the lymph nodes in four cases. Nerve‐sparing surgery is performed in 22 cases (Table 1).

**TABLE 1:** Patient characteristics (n = 114).

Characteristic	Value
Median (interquartile range) age at surgery (years)	68 (64–72)
Median (interquartile range) initial PSA level (ng/mL)	7.8 (5.4–10.6)
Median (interquartile range) prostate volume (ml)	27.0 (22.2–40.2)
Median (interquartile range) time from MRI to surgery	115 (83–169)
Medical History	
Diabetes mellitus	20
Hypertension	41
Myocardial infarction	3
Neurological disorders	4
Pathological TNM classification at surgery	
T2a	22
T2b	13
T2c	54
3a	12
3b	13
N1	4
Gleason score at the diagnosis of prostate cancer	
6	17
7	58
8	24
9	14
10	4
Number of cases with nerve‐sparing	22
Number of cases with lymph node dissection	58

**FIGURE 3:**
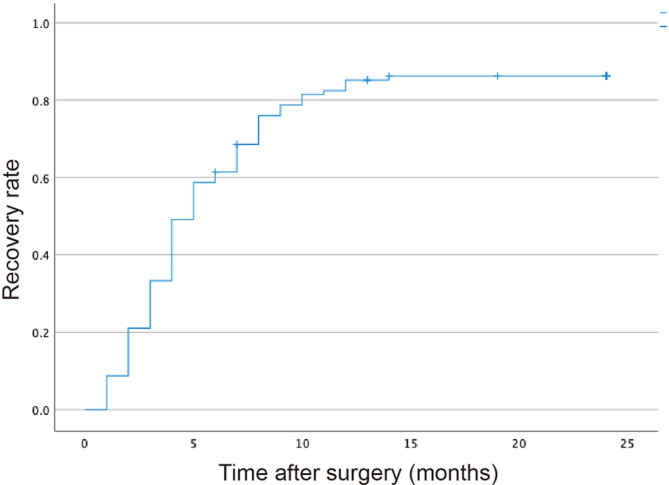
Cumulative recovery rate of urinary continence after robot‐assisted radical prostatectomy (RARP). The x‐axis represents the time (months) following surgery, while the y‐axis indicates the cumulative recovery rate. The median time to urinary continence recovery was 5 months, with a continence resolution rate of 85.1% during the observation period. Each step on the graph represents the number of patients who achieved urinary continence at different time points postoperatively.

The median time to incontinence recovery was 5.0 months (95% confidence interval: 4.2–5.7 months), with an incontinence resolution rate of 85.1% during the observation period (Figure 3). The patients were categorized into two groups: those who achieved continence earlier than the median (early group, n = 77) and those who did not (delayed group, n = 47), and a comparative analysis was performed.

The median age of the patients was 68 years (IQR: 64–72 years) in the early group and 68 years (IQR: 64–71 years) in the delayed group, with no significant difference (p = 0.43) (Table 2). The median PSA level at diagnosis was 7.2 ng/ml (IQR: 5.1–10.4 ng/ml) in the early group and 8.05 ng/ml (IQR: 5.7–10.9 ng/ml) in the delayed group, with no significant difference (p = 0.50). The median prostate volume was 26.6 ml (IQR: 23–35 ml) in the early group and 29 ml (IQR: 19.5–40 ml) in the delayed group, with no significant difference (p = 0.59).

**TABLE 2:** Comparison between early and late recovery cases.

	Early (n = 67)	Late (n = 47)	p‐value
Median (IQR) age at surgery (years)	68 (64–72)	68 (64–71)	0.4
Median (IQR) initial PSA level (ng/mL)	7.2 (5.1–10.4)	8.05 (5.7–10.9)	0.5
Median (IQR) prostate volume (ml)	26.6 (23–35)	29 (19.5–40)	0.5
Diabetes mellitus	13	7	0.6
Hypertension	27	14	0.3
Myocardial infarction	1	2	0.5
Neurological disorders	2	2	1.0
Prior anti‐androgen deprivation therapy (N)	17	11	1.0
Median (IQR) obturator internus muscle (mm)	18.7 (17.4–19.6)	17.3 (15.9–19.0)	0.025
Median (IQR) pubococcygeus muscle (mm)	8.2 (7.5–8.8)	7.5 (7.1–8.2)	0.004
Median (IQR) puborectalis muscle (mm)	5.9 (5.4–6.7)	5.9 (5.1–6.4)	0.3
Pathological T3 or more (N)	15	10	1.0
N1 (N)	3	1	0.6
Gleason score of 8 or higher in the resected specimen (N)	18	21	0.071
Nerve‐sparing (N)	18	4	0.016
Lymph node dissection (N)	33	25	0.7
Salvage radiation therapy after the surgery (N)	7	7	0.5

Continuous variables are presented as medians with interquartile ranges (IQR) and compared using the Mann–Whitney U test. Categorical variables are presented as counts (N) and compared using Chi‐squared test.

Comorbidities, including hypertension, diabetes, myocardial infarction and neurological disorders, are not significantly different between the early and delayed groups (p = 0.62, 0.32, 0.56 and 1.0, respectively) (Table 2). There is also no significant difference in the use of preoperative androgen deprivation therapy between the two groups (p = 1.0).

As for muscle diameters, the median diameter of the obturator internus muscle is 18.7 mm (IQR: 17.4–19.6 mm) in the early group and 17.3 mm (IQR: 15.9–19.0 mm) in the delayed group, with a significant difference observed (p = 0.025). The median diameter of the pubococcygeus muscle was 8.2 mm (IQR: 7.5–8.8 mm) in the early group and 7.5 mm (IQR: 7.1–8.2 mm) in the delayed group, also showing a significant difference (p = 0.004). No significant differences were observed in other pathological indicators, such as pathological stage T3 or higher (p = 1.0) or the presence of lymph node metastasis (N1) (p = 0.64). However, high‐grade cancer with a Gleason score of 8 or higher was more frequent in the delayed group (p = 0.071), and nerve‐sparing cases were more common in the early group (p = 0.016).

Based on these results, the cutoff values for the obturator internus and pubococcygeal muscles were set at 17.15 mm and 8.27 mm, respectively. Initially, univariate Cox proportional hazards regression analysis was conducted to identify potential predictors of incontinence recovery. Variables demonstrating statistical significance in the univariate analysis, including the obturator internus muscle width (≥ 17.15 mm), pubococcygeus muscle width (≥ 8.27 mm), nerve‐sparing status and puborectalis muscle width, were subsequently incorporated into multivariate analysis (Table 3). Cox proportional hazards multivariate analysis revealed that both the obturator internus and pubococcygeus muscles are independent and significant prognostic factors for incontinence recovery (Table 3).

**TABLE 3:** Univariate and multivariate Cox hazard analyses for the timing of incontinence improvement.

Variable	Univariate					Multivariate				
	B	SE	Wald	Sig.	Exp(B)	B	SE	Wald	Sig.	Exp(B)
Age	−0.03	0.01	3.32	0.068	0.9					
Initial PSA level	<0.001	0.01	<0.001	0.9	1					
Prostate volume	−0.004	0.005	0.6	0.4	0.9					
Diabetes mellitus with vs. without	0.11	0.26	0.17	0.6	1.1					
Hypertension with vs. without	0.06	0.21	0.08	0.7	1					
Myocardial infarction with vs. without	−0.48	0.71	0.45	0.5	0.6					
Neurological disorders with vs. without	0.2	0.51	0.16	0.6	1.2					
Prior anti‐androgen deprivation therapy with vs. without	0.11	0.23	0.24	0.6	1.1					
Obturator internus muscle width ≥ 17.15 mm	0.78	0.23	10.86	0.001	2.1	0.67	0.25	7.09	0.0077	1.9
Pubococcygeus muscle width ≥ 8.27 mm	0.71	0.21	11.45	0.0007	2	0.66	0.21	8.87	0.0022	1.9
Puborectalis muscle	0.21	0.08	5.64	0.017	3.7	0.07	0.09	0.76	0.3	1
Pathological T3 or more with vs. without	−0.18	0.25	0.52	0.4	0.8					
N1 with or without	0.25	0.51	0.24	0.6	1.2					
Gleason score of 8 or higher with vs. without	−0.28	0.21	1.77	0.18	0.7					
Nerve‐sparing with vs. without	0.56	0.24	5.17	0.022	1.7	0.47	0.25	3.54	0.059	1.6
Lymph node dissection with vs. without	−0.14	0.2	0.53	0.4	0.8					
Salvage radiation therapy with vs. without	−0.32	0.3	1.08	0.29	0.7					

Univariate analysis evaluated each variable independently, whereas multivariate analysis incorporated all variables simultaneously to account for potential confounding factors. The results are presented as regression coefficients (B), standard errors (SE), Wald chi‐squared values (Wald), significance levels (Sig.) and hazard ratios (Exp(B)).

## 4 DISCUSSION

The primary objective of this study was to identify the key factors influencing early continence recovery in patients who underwent RARP, with a particular focus on the impact of preoperative pelvic floor muscle thickness. A retrospective analysis of the institutional data was performed to assess patient characteristics, perioperative variables and surgical outcomes. The median postoperative time to continence recovery was 5 months, with an incontinence resolution rate of 85.1% during the observation period. According to previous reviews and meta‐analyses, the 12‐month post‐RARP incontinence rate ranges from 4% to 31%, with an average of 16%, which is consistent with the findings of this study.^4^ Multiple surgical factors are known to influence postoperative urinary continence recovery, including nerve‐sparing techniques, dorsal venous complex (DVC) ligation, reconstruction methods and surgeon experience.^11^ All nerve‐sparing procedures in this study utilized the intrafascial nerve‐sparing technique. The ligation and division of the DVC were performed uniformly in all cases by the same surgeon. Among the surgeons, two routinely performed anterior reconstruction in addition to Rocco posterior reconstruction, whereas all other surgeons did not. A statistical analysis was conducted to evaluate the impact of anterior reconstruction on early incontinence recovery; however, no significant difference was observed (early n = 43, late n = 37; p = 0.09). Furthermore, Retzius‐sparing and hood techniques were not employed in any of the surgical procedures conducted in this investigation. During the study period, surgeries were performed by three highly experienced surgeons and two relatively less experienced surgeons, with the Rocco stitch applied in all cases. The number of cases performed by less experienced surgeons was small (early, n = 4; late, n = 4), resulting in no significant difference in continence recovery outcomes between experienced and less experienced surgeons (p = 0.60). These data suggest that the majority of surgical factors were standardized across the cases in this study; consequently, their influence on continence recovery outcomes appears to be minimal. A comparison between the early and delayed recovery groups revealed that age, malignancy grade, prior anti‐androgen deprivation therapy (ADT), lymph node dissection and use of postoperative salvage radiotherapy did not significantly affect the time to continence recovery. The administration of ADT prior to RARP was determined at the discretion of the attending physician and was not primarily intended for disease downstaging. Instead, ADT was frequently administered to patients who initially selected hormonal therapy or combined hormonal and radiation therapy but subsequently altered their preference to surgery. Moreover, ADT was utilized to mitigate disease progression during the preoperative waiting period. No significant difference was observed in the number of patients receiving preoperative ADT between the early and late recovery groups. Additionally, the duration of ADT did not differ significantly between the groups, with a median duration of 58 days (IQR: 48–128) in the early recovery group (n = 15) and 56 days (IQR: 49–62) in the late recovery group (n = 11) and a p‐value of 0.6. The relatively brief duration of ADT, approximately two months, may have contributed to the absence of observed influence on the timing of urinary continence recovery. Among the 14 patients who underwent salvage radiation therapy, continence improvement occurred after radiation in 8 patients. The distribution was even between the early (n = 4) and late (n = 4) recovery groups. This finding suggests that the timing of RT relative to continence recovery did not have a significant impact on the outcomes. However, cases in which nerve sparing was performed revealed a significantly higher rate of early continence recovery.^12^ Furthermore, the thicknesses of the obturator internus and pubococcygeus muscles were significantly greater in the early recovery group, suggesting that pelvic floor muscle thickness, along with nerve sparing, significantly impacted early postoperative incontinence recovery. In the multivariate analysis, both pelvic floor muscles were identified as independent predictors. The significance of levator ani muscle thickness in early urinary continence recovery following RARP has been previously demonstrated.^13^ This investigation extends previous findings by examining individual muscles within the levator ani complex, specifically the pubococcygeus and puborectalis muscles, to provide a more comprehensive understanding of their respective roles in continence recovery. Furthermore, this study emphasizes the significance of the obturator internus muscle, which is not a component of the levator ani group, as a crucial factor in urinary continence recovery. To the best of our knowledge, this research represents one of the initial studies to propose the clinical relevance of obturator internus muscle thickness in this context. These results indicate that pelvic floor muscle evaluation should include not only the levator ani but also surrounding muscles, such as the obturator internus, for a more comprehensive assessment.

The urethra is surrounded by the urethral sphincter, a smooth muscle layer and pelvic floor muscles (the pubococcygeus, puboperineal and puborectalis muscles), which support the urethra and control urination. These muscles are innervated by nerves, and nerve‐sparing likely preserves the nerves essential for regulating these muscles. The pubococcygeus muscle is a key component of the pelvic floor muscle group, contributing to the maintenance of urinary and bowel function, and is also associated with pelvic organ prolapse (POP).^14^, ^15^ Regarding the pubococcygeus muscle in RARP cases, previous studies examining the correlation between intraoperative injury and postoperative recovery have not yielded significant results, likely because intraoperative injuries were extremely rare. However, in our study, the analysis was based on preoperative evaluations, which likely explains the differing results.^9^

A previous study investigating the relationship between POP in women found that the pubococcygeal muscle correlates with the severity of POP, suggesting that it is a crucial muscle for supporting the pelvic floor, regardless of sex.^16^ In contrast, the obturator internus, located on the lateral wall of the pelvis plays a role in supporting the pelvic floor via the obturator fascia and is involved in the external rotation of the hip joint.^17^, ^18^ Strengthening the obturator internal muscles can improve pelvic floor muscle function. As the obturator internus muscle has extensive contact with the levator ani, including the pubococcygeus muscle, its movement has been revealed to influence the levator ani, impacting both defecation and urinary function. Rehabilitation and strengthening of the obturator internus could contribute to enhancing pelvic floor muscle function and may also be effective in improving urinary function.^19^ Given the role of the obturator internus in external hip rotation, incorporating external rotation exercises into preoperative pelvic floor training may enhance muscle strength and improve postoperative continence recovery. This approach is particularly relevant in older patients, in whom age‐related muscle atrophy is more prevalent.

In addition to examining muscle thickness, we also considered the impact of ageing on urinary incontinence improvement recovery. Previous reports have shown that the rate of postoperative incontinence improvement exceeds 90% in patients aged 60, whereas it remains at 69% in those aged 70 and older, indicating that age may significantly affect urinary continence.^2^ In this study, a significant correlation was found between the thickness of the obturator internus muscle (Spearman's rank correlation coefficient: −0.37, p < 0.0001) and nerve‐sparing (−0.35, p < 0.0001), while no significant association was observed for the pubococcygeus muscle. This suggests that age‐related lower limb muscle weakness and atrophy may affect the obturator internus muscle, potentially leading to dysfunction in coordination with the pubococcygeus muscle. However, this study has limitations. First, the small sample size and retrospective design warrant further prospective research. Additionally, data on urinary continence were based on self‐reported outcomes, posing a risk of selection bias. Future studies should include larger cohorts and use prospective designs.

## 5 CONCLUSIONS

These findings highlight the importance of preoperative pelvic floor muscle training and patient education, particularly in older patients. Such training should be recognized as a standard component of surgical planning. Incorporating external rotation exercises targeting the obturator internus into preoperative regimens may further enhance pelvic floor strength, potentially leading to improved postoperative outcomes. Moreover, variables such as obturator internus and pubococcygeus muscle thickness, as well as nerve‐sparing status, may potentially serve as a foundation for developing scoring systems to facilitate preoperative counselling. Further investigation is warranted to validate these findings and explore their integration into practical clinical tools.

## AUTHOR CONTRIBUTIONS

Conception: SH, DO and ST. Data collection: HU, YI. Interpretation of data: JM, TY, KY and ST. Writing the article: SH, DO. All authors had access to the data.

## CONFLICT OF INTEREST STATEMENT

The authors have no conflicts of interest to report.

## Data Availability

Data supporting the findings of this study are available from the corresponding author upon request.
